# miR-22 and cerebral microbleeds in brainstem and deep area are associated with depression one month after ischemic stroke

**DOI:** 10.1590/1414-431X20209162

**Published:** 2020-04-27

**Authors:** Jia Hu, Wei Zhou, Zhiming Zhou, Qian Yang, Junfeng Xu, Wanli Dong

**Affiliations:** 1 Department of Neurology, The First Affiliated Hospital of Soochow University, Suzhou, Jiangsu, China; 2 Department of Neurology, The First Affiliated Hospital, Yijishan Hospital of Wannan Medical College, Wuhu, Anhui, China; 3 Department of Cardiovascular surgery, The First Affiliated Hospital, Yijishan Hospital of Wannan Medical College, Wuhu, Anhui, China

**Keywords:** Depression, microRNA, Stroke, Cerebral microbleeds, Magnetic resonance imaging

## Abstract

In this study, we aimed to explore the relationship among miR-22, deep cerebral microbleeds (CMBs), and post-stroke depression (PSD) 1 month after ischemic stroke. We consecutively recruited 257 patients with first-ever and recurrent acute cerebral infarction and performed PSD diagnosis in accordance with the Diagnostic and Statistical Manual IV criteria for depression. Clinical information, assessments of stroke severity, and imaging data were recorded on admission. We further detected plasma miR-22 using quantitative PCR and analyzed the relationship among miR-22, clinical data, and PSD using SPSS 23.0 software. Logistic regression showed that deep (OR=1.845, 95%CI: 1.006-3.386, P=0.047) and brain stem CMBs (OR=2.652, 95%CI: 1.110–6.921, P=0.040), as well as plasma miR-22 levels (OR=2.094, 95%CI: 1.066–4.115, P=0.032) were independent risk factors for PSD. In addition, there were significant differences in baseline National Institutes of Health Stroke Scale scores (OR=1.881, 95%CI: 1.180–3.011, P=0.007) and Widowhood scores (OR=1.903, 95%CI: 1.182–3.063, P=0.012). Analysis of the receiver operating curve (AUC=0.723, 95%CI: 0.562–0.883, P=0.016) revealed that miR-22 could predict PSD one month after ischemic stroke. Furthermore, plasma miR-22 levels in brainstem and deep CMBs patients showed an upward trend (P=0.028) relative to the others. Patients with acute ischemic stroke, having brainstem and deep cerebral microbleeds, or a higher plasma miR-22 were more likely to develop PSD. These findings indicate that miR-22 might be involved in cerebral microvascular impairment and post-stroke depression.

## Introduction

Post-stroke depression (PSD) is an emotional disorder and one of the most common complications of ischemic stroke. The occurrence of PSD is always followed by negative clinical outcomes including cognitive and functional impairment. Furthermore, the condition is a prominent barrier to stroke rehabilitation, contributing to reduced quality of life and increased risk of stroke and mortality (1). Given that its pathogenesis is still unclear, there is a need to study risk factors related to PSD and further explore new stable biomarkers for its diagnosis and treatment.

MicroRNAs (miRNAs) are small non-coding RNAs of about 19–25 nucleotides long that bind onto mRNA 3′ untranslated region (UTR) and regulate mRNA degradation as well as inhibit protein synthesis (2). Previous studies have reported that miRNAs are involved in regulating endothelial dysfunction, cell proliferation and apoptosis, inflammatory response, oxidative stress, angiogenesis, and neurogenesis (3). At the same time, they play an important role in release of neurotransmitters and synaptic plasticity (4). Moreover, these RNAs have been shown to play a critical role in major depression and suicidal behavior, generating a negative outcome (5). Circulating miRNAs have been identified as novel potential blood biomarkers in some mental diseases including major depression. Previous studies have shown that differential expression of miRNAs influenced homeostasis of neural and synaptic pathways, by negatively regulating expression of genes such as brain-derived neurotrophic factor (*BDNF*)-tyrosine kinase receptor B (*TrkB*) pathways and other important signaling pathways (6,7). Our previous research showed that tyrosine kinase receptor B was associated with PSD in Chinese people (8).

A search through miRNAs target genes online prediction software for miRNAs related to *TrkB* revealed that miR-22 is strongly associated with NTRK2. Meanwhile, previous research using gene chip has shown that miR-22 is just one of 25 differentially expressed miRNAs between PSD and non-PSD patients (9), although its diagnostic efficacy and possible mechanisms of action remain unknown. miR-22 has also been reported to be involved in pathophysiological processes such as endothelial dysfunction. Overexpressing miR-22 was found to induce inflammation, damage endothelial cells, increase vascular permeability, and finally destroy the blood-brain barrier (10). Dysfunction of endothelial cells and destruction of the blood-brain barrier were involved in the pathophysiology of microbleeds (11). In the current study, we hypothesized that cerebral micro-vascular impairment might be one of the targets of miR-22 driving PSD.

To our knowledge, this is the first study to demonstrate a relationship between post-stroke depression and miR-22, as well as its clinical indicators. The aim of the study, therefore, was to clarify the role of miR-22 in the pathogenesis of PSD. We also sought to confirm whether miR-22 can serve as a novel biomarker and a potential therapeutic target of PSD.

## Material and Methods

### Study subjects

This study enrolled 351 patients diagnosed with acute cerebral infarction following magnetic resonance imaging (MRI) scans performed at the Department of Neurology, Yijishan Hospital from March 2015 to September 2017. Inclusion criteria were as follows: i) all enrolled patients met the criteria defined by the World Health Organization Multinational Monitoring of Trends and Determinants in Cardiovascular Disease (WHO-MONICA) for acute ischemic stroke (AIS), and were verified by MRI within 24 h of admission; ii) the course of disease was less than 1 week; and iii) patients were over 18 years of age. Exclusion criteria included the following: i) patients with psychiatric conditions such as depression; ii) patients with unconsciousness, dementia, or significant cognitive impairment; iii) patients with severe aphasia or dysarthria, visual or auditory impairment; iv) patients with metabolic abnormalities, tumors, significant acute inflammatory disease, or other medical illness besides stroke; and v) patients on whom MRI scans were not performed for various reasons. All patients were followed up for 1 month. A total of 257 patients completed the follow-up and were eventually enrolled in the study. In addition, the study ensured that each patient's clinical symptoms met the diagnostic criteria for cognitive impairment and dementia outlined in the “Chinese Guidelines for Diagnosis and Treatment of Cognitive Impairment and Dementia” (http://www.chinadoi.cn/portal/mr.action?doi=10.3760/cma.j.issn.0376-2491.2010.41.003). Patients with MMSE≤9 were considered to have severe cognitive impairment or dementia.

The study was approved by the Ethics Committee of First Affiliated Yijishan Hospital of Wannan Medical College. Written informed consents were signed by participants or their relatives before including them in the study.

### Methods

*Collection of clinical data.* Baseline clinical data collected from the enrolled subjects included demography (age, sex, body mass index, and education), vascular risk factors (hypertension, diabetes mellitus, hyperlipidemia, and coronary heart disease), as well as medical history (smoking, alcohol consume, and cases of previous stroke). Stroke subtypes were classified according to criteria outlined by TOAST (12) while its severity was evaluated by trained neurologists using the National Institutes of Health Stroke scale (NIHSS) within 24 h of admission. The Hamilton Depression-17 (HAMD-17) scale (13) and clinical interviews were performed by a trained neurologist one month after onset of stroke (Qian Yang). The HAMD-17 scale was also used to assess depressive symptoms one month after onset while diagnosis of PSD was done in accordance with Diagnostic and Statistical Manual IV criteria for depression one month after stroke (14).

### Assessment of imaging data

Head MRI scans (3.0t GE Multisync LCD, USA) were performed within 1 week of admission. Briefly, neuroimaging markers of small vessel disease were assessed using STRIVE nomenclature by analyzing MR images. Scan sequence included T1 and T2 weighted imaging, sequence of fluid attenuated inversion recovery (FLAIR), diffusion weighted imaging (DWI), magnetic resonance angiography (MRA), and susceptibility-weighted imaging (SWI). The parameters used included 3.0t MRI SWI: TR 76.1 ms, TE 42.58 ms, slice thickness/gap 2/1 mm, 3.0t DWI TR 3000 ms, TE 65.5 ms, slice thickness/gap 6/7 mm; MRA TR 21 ms, TE 2.5 ms, and slice thickness/gap 1.4/0.7 mm. Based on the STRIVE nomenclature (15), imaging data was divided into the following aspects: enlarged perivascular spaces, white matter hyperintensities, cerebral microbleeds, lacunar cerebral infarction, and recent small subcortical infarction.

Cerebral microbleeds (CMB) were defined as multiple ovoid foci with marked loss of signal intensity on T2*-weighted, gradient-recalled echo MRI. SWI showed low round point signal with a diameter of 2∼10 mm, at least half of which was surrounded by brain parenchyma with no edema. Symmetrical basal ganglia calcification, vascular cavitation effect, cavernous hemangioma, and diffuse axonal injury were excluded. Lesions located within the infarct area were not considered microbleeds. In addition, CMBs were divided into lobar, deep, and posterior fossa (brain stem and cerebellum) groups while white matter hyperintensities on MRI were defined as hyperintensities ≥5 mm on T2 and T2FLAIR images. Severity of white matter hyperintensities was assessed using the Fazekas scale (0–3 points). White matter hyperintensities (WMHs) were divided into periventricular (PVWMHs) and deep (DWMHs). Meanwhile, MRI was used to locate infarction lesions and count the number of acute infarctions in the cortex, subcortical white matter, deep, and sub-tentorial.

### Selection and detection of miRNAs

According to our previous research, tyrosine kinase receptor B is strongly associated with PSD in Chinese people. In this study, we searched for miRNAs related to the *TrkB* gene (NTRK2, gene number: ENSG00000148053) in TargetScan; an online software for prediction of microRNA target genes (http://www.targetscan.org/) and miRBD (http://mirbd.org/miRBD/Index.html). An intersection between results from the two databases showed that miR-22 was most strongly related to *NTRK2*. At the same time, we compared this with previous results of miRNAs gene chip in post-stroke depression (9) and found that miR-22 was one of 25 miRNAs that showed a significant differential expression in patients with post-stroke and non-post-stroke depression.

In both groups, we selected 20 (from a total of 40 patients) for each group for detection of plasma miR-22. We ensured that there were no significant differences in gender, age, stroke site, NIHSS score, and TOAST type between the two groups (P>0.05).

In the morning of the day after admission, 4 mL of venous blood was collected into an EDTA anticoagulant tube. The blood sample was centrifuged at 800 *g* for 15 min at 4°C within 2 h of collection, the upper plasma was encapsulated in EP tubes, and then frozen at -80°C before measurement. Total plasma RNA was isolated and purified using the mirVanaTM RNA Isolation kit (Applied Biosystem p/n AM1556, USA). Concentration of each total RNA sample was determined using a Nanodrop 2000 spectrophotometer (Thermo, USA). Each sample was tested 3 times and those with an A260/A280 value between 1.9 and 2.2 were used in subsequent experiments. RNA was reverse transcribed using the miScript reverse transcription kit (Qiagen; 218161, Germany) according to the manufacturer's instructions. A synthetic *C. elegans* miR-39 (cel-miR-39, Qiagen; 219610), which is devoid of sequence homology to human microRNAs, was included into the plasma after lysis as an external reference. Primers were designed using Primer 5.0 software according to GenBank and miRBASE databases (Table 1), then synthesized by Shanghai Shengong Biotechnology Co. Ltd. (China). To determine expression of the miRNAs, we performed a real-time PCR using the miScript SYBR Green PCR kit (Qiagen; 218073) according to the manufacturer's instructions and each PCR was repeated 3 times. Melting curves were analyzed using the software in the fluorescence quantitative PCR machine and Ct-values generated. Calculation of miR-22 expression was conducted by 2^-△△Ct^ relative quantification method, using *cel-miR39* as the internal reference gene (16).

**Table 1:** Primer sequences for quantitative real time polymerase chain reaction reverse transcription.

Primer	Sequence (5′ to 3′)
qPCR-miR-22-F	TGCGGAGTTCTTCAGTGGCAA
qPCR-miR-22-R	CAGTGCAGGGTCCGAGGT
qPCR- Cel-miRNA-39-F	CGTCGATCACCGGGTGTAAA
qPCR- Cel-miRNA-39-R	CTCTGTCTCTCGTCTTGTTGGTAT

### Data analysis

SPSS version 23.0 statistical software for data processing and analysis was used. The Kolmogorov-Smirnov test was first used for evaluating normality of the data. Data showing a normal distribution are reported as means±SD, with an independent sample *t*-test used for comparison between groups. Data with non-normal distribution are reported as median and interquartile range. Comparisons between groups were conducted by the Mann-Whitney U-test, while comparisons among multiple groups were done using the Kruskal-Wallis test. Count data are reported as frequencies and percentages. A multiple logistic regression analysis was used to evaluate PSD-related factors by calculating odds ratio (OR) and 95% confidence interval (CI). A Pearson correlation was used to generate a relationship among variables. Finally, the receiver operating curve (ROC) was drawn to analyze the diagnostic efficacy of miR-22 at P≤0.05 significance level.

## Results

### General patient data

**Figure 1:**
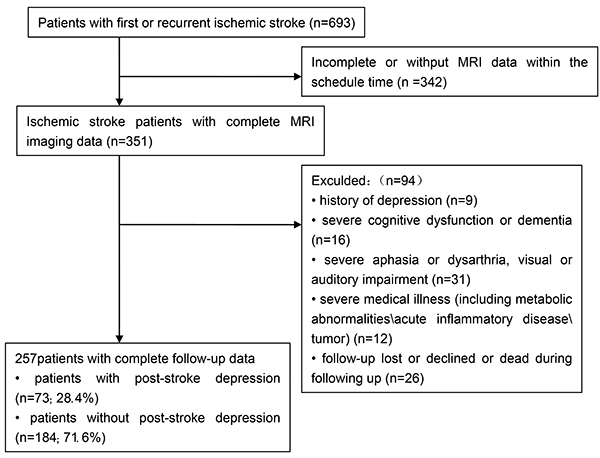
Study recruitment profile.

Of the 257 patients enrolled in the study, 151 (58.8%) were male and 106 (41.2%) were female. The average age of these patients was 62.3±10.7 years (Figure 1). Excluded patients (n=436) had no significant difference in gender (48.4 *vs* 41.2%, P=0.068) relative to the enrolled patients (n=257). On the other hand, significant differences were noted in age (64.8±11.5 *vs* 62.3±10.7, P=0.005) and severity of stroke (NIHSS score 6.5±5.3 *vs* 5.1±4.2, P<0.001). In the enrolled group, depression was noted in 73 (28.4%) of the patients while the other 184 (71.6%) showed no symptom one month after stroke. Basic characteristics of the 257 patients with and without post-stroke depression are reported in Table 2. In summary, patients in the PSD and non-PSD groups showed no difference with regards to age, gender, history of stroke, common vascular risk factors of ischemic stroke (such as hypertension, hyperlipidemia, diabetes, coronary heart disease, smoking, and drinking) as well as TOAST classification of stroke etiology. On the other hand, significant differences were recorded in the groups with regards to education (P=0.027), widowhood (P=0.002), BMI levels (P=0.033), and baseline NIHSS scores P<0.001).

**Table 2:** Baseline clinical characteristics of patients with and without post-stroke depression (PSD) at 1 month.

Variables	PSD group (n=73)	Non-PSD group (n=184)	t/Z/X^2^	P value
Demographic characteristics				
Age (y, mean±SD)	62.7±10.1	62.1±10.3	0.423	0.672
Female (n, %)	34 (46.60)	72 (39.1)	1.195	0.274
Educational (y, mean±SD)	4.6±3.4	5.6±3.2	−2.219	0.027
BMI (kg/m^2^, mean±SD)	24.9±2.5	24.2±2.3	2.146	0.033
Widowhood (n, %)	24 (32.9)	29 (15.8)	9.353	0.002
Previous stroke (n, %)	19 (26.0)	40 (21.7)	0.543	0.461
Vascular risk factors (n, %)				
Hypertension	61 (83.6)	134 (72.8)	3.291	0.069
Hyperlipidemia	31 (42.5)	71 (38.6)	0.329	0.567
Diabetes mellitus	32 (43.8)	73 (39.7)	0.345	0.541
Coronary heart disease	14 (19.2)	25 (13.6)	1.269	0.260
Active smokers	30 (41.1)	72 (39.1)	1.146	0.285
Alcohol consumption	21 (28.8)	44 (23.9)	0.420	0.652
Type of stroke etiology (n, %)			0.268	0.605
Atherothrombotic	24 (32.9)	61 (33.2)		
Lacunar	16 (21.9)	33 (17.9)		
Cardioembolic	18 (24.7)	43 (23.4)		
Others	15 (20.5)	47 (25.5)		
Baseline NIHSS score (median, IQR)	9 (7-12)	8 (6-11)	7.321	0.001

BMI: body mass index; NIHSS: National Institutes of Health Stroke Scale; IQR: interquartile range; SD: standard deviation. Data were analyzed by the *t*-test, Mann-Whitney U-test, or chi-square test.

### Brain stem and deep CMBs were associated with PSD

Patients in the PSD group were more likely to have brain stem (17.8 *vs* 7.6%; P=0.023) and deep CMBs (28.8 *vs* 16.8%; P=0.032) compared to those in the non-PSD group (Table 3). We found no significant difference between PSD and the non-PSD groups with regards to Fazekas PVWMHs and Fazekas DWMHs scores, CMBs in other parts of the brain except frontal lobe, brainstem, and deep area, as well as the number, size, location, or laterality of acute infarction lesions.

**Table 3:** Imagining characteristics in patients with and without post-stroke depression (PSD) at 1 month.

Variate	PSD group (n=73)	Non-PSD group (n=184)	t/Z/X^2^	P value
EPVS (median, IQR)	1 (1-2)	1 (0-2)	0.847	0.380
Fazekas DWMHs score (median, IQR)	1 (1-2)	1 (1-2)	0.738	0.460
Fazekas PVWMHs score (median, IQR)	1 (1-1)	1 (0-1)	0.981	0.327
Number of CMBs (mean±SD)	9.5±6.1	9.7±4.8	-0.255	0.799
Location of CMBs (n, %)				
Lobar	27 (37.0)	64 (34.8)	0.111	0.739
Deep	21 (28.8)	31 (16.8)	4.601	0.032
Brainstem	13 (17.8)	14 (7.6)		0.023
Cerebellum	3 (4.1)	12 (6.5)		0.566
Lacure (n, %)	21 (28.8)	41 (22.3)	1.201	0.273
Number of acute ischemic cerebral infarction (median, IQR)	1 (1-1)	1 (1-1)	-0.323	0.747
Location of acute infarct (n, %)			0.210	0.647
Cortical	17 (23.3)	42 (22.8)		
Subcortical white matter	7 (9.6)	20 (10.9)		
Deep	28 (38.4)	81 (44.0)		
Infra tentorial	21 (28.8)	41 (22.3)		
Stroke characteristics (%)			1.262	0.261
Small infarct (<15 mm)	25	77		
Large infarct (≥15 mm)	48	107		
Lateralization (n, %)			0.938	0.333
Left hemisphere	29 (39.7)	83 (45.1)		
Right hemisphere	32 (43.8)	78 (42.4)		
Bilateral hemisphere	12 (16.4)	23 (12.5)		

EPVS: enlarged perivascular spaces; DWMHs: deep white matter hyperintensities; PVWMHs: periventricular white matter hyperintensities; CMBs: cerebral microbleeds; AIS: acute ischemic stroke; IQR: interquartile range; SD: standard deviation. Data were analyzed by the *t*-test, Mann-Whitney U-test, Kruskal-Wallis test, or chi-square test.

We incorporated levels of the biomarker miR-22 into a logistic regression and performed an adjusted logistic regression. Results showed that deep (OR=1.845, 95%CI: 1.006–3.386, P=0.047) and brain stem CMBs (OR=2.652, 95%CI: 1.110–6.921, P=0.040) were significant predictors. In addition, the plasma level of miR-22 (OR=2.094, 95%CI: 1.066–4.115, P=0.032) was statistically related to PSD. Other predictors included baseline NIHSS (OR=1.881, 95%CI: 1.180–3.011, P=0.007) and widowhood (OR=1.903, 95%CI: 1.182–3.063, P=0.012) (Table 4).

**Table 4:** Multivariate logistic model of the clinical determinants of post-stroke depression.

Variables	OR (95%CI)	P value
Brainstem CMBs	2.652 (1.110−6.921)	0.040
Deep CMBs	1.845 (1.006−3.386)	0.047
Years of educational	0.867 (0.669−1.124)	0.282
BMI	1.432 (0.987−2.061)	0.058
Widowhood	1.903 (1.182−3.063)	0.012
Baseline NIHSS score	1.881 (1.180−3.011)	0.007
Plasma miR-22 level	2.094 (1.066−4.115)	0.032

CMBs: cerebral microbleeds; BMI: body mass index; NIHSS: National Institutes of Health Stroke Scale.

### Correlation between plasma miR-22 expression and post-stroke depression

**Figure 2:**
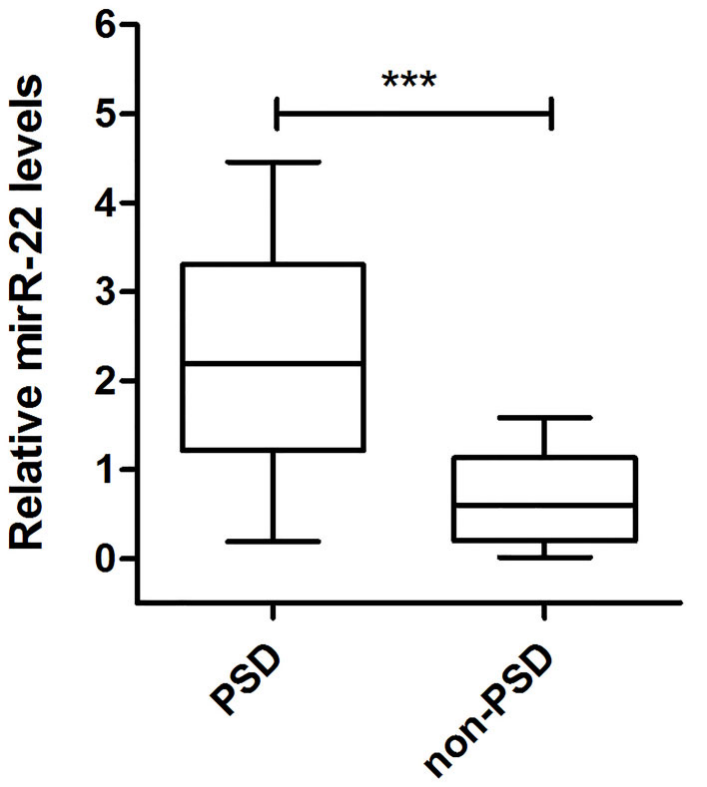
Relative expression of miR-22 in plasma compared between the post-stroke depression (PSD) group and the non-PSD group. Data are reported as median and interquartile range. ***P<0.001 (Mann-Whitney U-test).

Analysis of plasma miR-22 level showed an upward trend in subjects in the PSD group relative to those in the non-PSD group (P<0.0001) (Figure 2).

**Figure 3:**
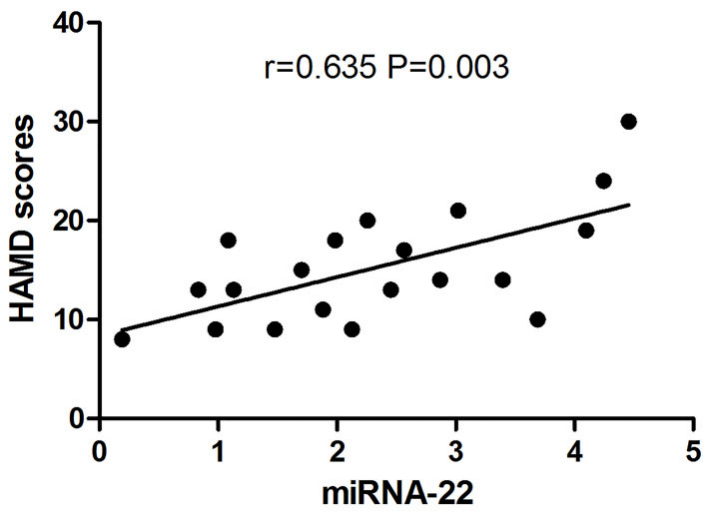
Pearson’s correlation analysis between the relative expression of plasma miR-22 and Hamilton depression (HAMD) score in post-stroke depression patients.

Similarly, the Pearson's test showed that HAMD score was positively correlated with plasma miR-22 in post-stroke depression one month after stroke (r=0.635, P=0.003) (Figure 3).

**Figure 4:**
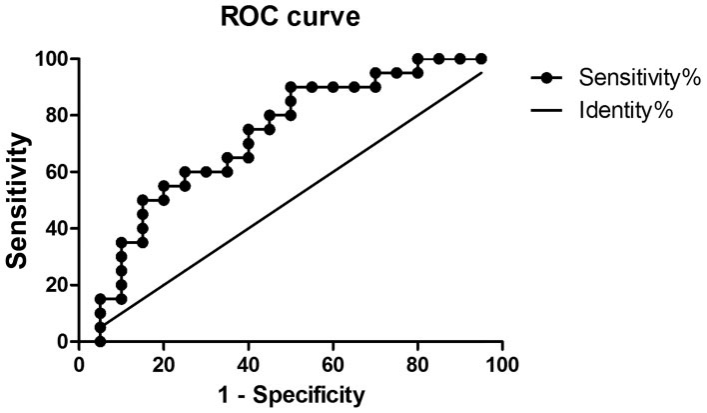
Receiver operating characteristic (ROC) curve demonstrating the predictive value of the relative expression of plasma miR-22 in post-stroke depression within 1 month.

Meanwhile, ROC curves showed that miR-22 could predict PSD one month after ischemic stroke. Specifically, an AUC of 0.723 (95%CI: 0.562–0.883, P=0.016), with a cut-off point of 0.90, 50% specificity, and 90% sensitivity were recorded (Figure 4). Plasma miR-22 concentrations above 0.90 at baseline represented a high risk of PSD.

**Figure 5:**
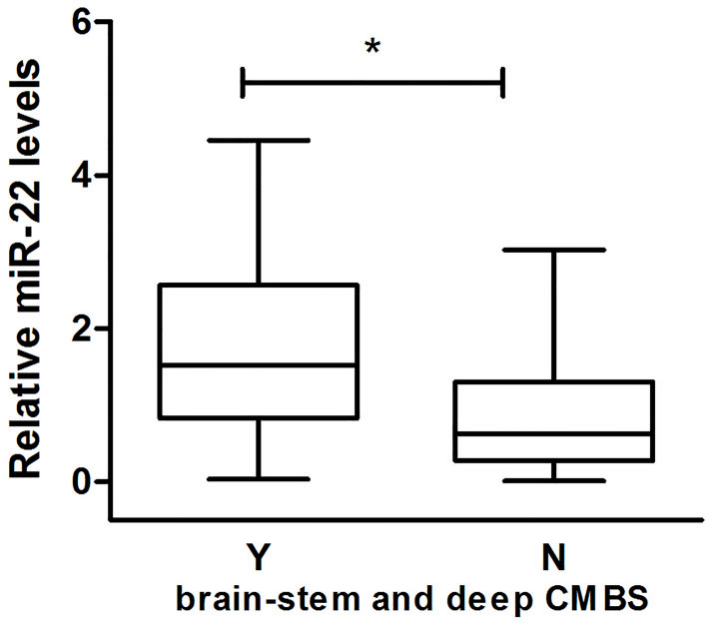
Boxplots comparison of the relative expression of miR-22 in patients with (Y) and without (N) cerebral microbleeds (CMBs) in brainstem and deep area. Data are reported as median and interquartile range. *P<0.05 (Mann-Whitney U-test).

A further analysis of the relationship between miR-22 and brainstem and deep CMBs showed an upward trend of plasma miR-22 in patients with brainstem and deep CMBs compared to those without (P=0.028) (Figure 5).

## Discussion

The findings of this study showed that acute ischemic stroke patients with brainstem and deep CMBs or a higher plasma miR-22 level were more likely to develop PSD. Specifically, we found that brain stem and deep CMBs were independent predictors of PSD. In addition, plasma miR-22 was related to PSD while miR-22 might be a new biomarker for the condition one month after stroke; taken together, these findings provided a new prospect for diagnosis and therapy of PSD.

CMBs, a type of cerebral vascular disease, have been implicated in the damage of early endothelial cells resulting in damage of the blood-brain barrier, neuroinflammation, and impairment of immune function (11). Different areas of CMBs play an important role in emotional control and executive function (17). In addition, damage to endothelial cells and blood-brain barrier have been reported to play an important role in CMBs, while vascular dysplasia might aggravate their pathological process. However, the underlying mechanisms of CMBs in depression are still unknown, although they might be explained by several pathways. Firstly, CMBs can cause structural disruptions of white matter tracts that are related to mood regulation and predispose an individual to depression (18). Secondly, microvascular dysfunction in subjects with CMBs is closely linked to chronic low-grade inflammation and/or oxidative stress (19). In fact, other studies have linked inflammation to depression or PSD via perfusion deficits or increased expression of serotonin receptors (20). Thirdly, CMBs might be associated with endothelial dysfunction, which could activate hemostatic mechanisms, inhibit reuptake of serotonin from the blood by platelets, reduce concentrations of intracellular serotonin, and eventually induce depression symptoms.

Serotonergic transmission deficiency is important in biological mechanisms during depression and PSD. Serotonergic innervations to the cerebral cortex and limbic structures originates almost exclusively from raphe nuclei in the caudal midbrain and rostral pons (21). Impaired serotonergic binding activities in these nuclei has been reported in PSD and major depression (22). In addition, structural disruptions in the brainstem raphe have consistently been found in depression and associated neuronal loss has further been reported in the same region (23). Meanwhile, deep brain CMBs, a kind of small cerebral vascular disease, reflect the disturbance of brain microcirculation with previous studies showing that chronic vascular burden might play an important role in PSD (24). Endothelial dysfunction and damage to the blood-brain barrier could lead to chronic hypoperfusion, which is related to chronic low-grade inflammation, and oxidative stress (19). Moreover, inflammation has been implicated in PSD through increased expression of serotonin receptors (20).

Current knowledge of microRNAs coupled with recent advancement in detection technology has increased research on miRNAs and stroke (25). Specifically, miRNAs are involved in and regulate many biological processes in the central nervous system, including endothelial dysfunction, apoptosis, cell proliferation, inflammatory response, oxidative stress, angiogenesis, and neurogenesis (26). Moreover, they are stable during blood circulation and have recently been identified as potential new types of biomarkers for treatment and diagnosis. Previous studies have shown that miRNAs play a role in the development of various mental disorders (27) with recent evidence implicating them in the occurrence, development, and treatment of depression (28).

In our previous study, we found a close relationship between *TrkB* and PSD (8). In the current study, we observed a correlation between miR-22 and *TrkB* gene (*NTRK2*) based on results from analysis of microRNAs target genes using an online prediction software. In addition, a significant differential expression was observed in miR-22 between patients with post-stroke and non-post-stroke depression following gene chip analysis (9). All these studies suggest that miR-22 might be a potential biomarker for the diagnosis of PSD. In our study, we also found a significant increase in plasma miR-22 in subjects from the PSD relative to those in the non-PSD group, which confirmed the results of the gene chip. A further evaluation of the diagnostic value indicated that MiR-22 might be a new blood biomarker for PSD one month after stroke.

*BDNF* has proven to be an important neurotrophic factor that affects neuron proliferation, synaptic function, and plasticity by binding to the receptor complex formed by *TrkB* (29). Activation of *TrkB* has been shown to result in phosphorylation of self-residues and sequential activation of intracellular cascade signals, including *MAPK/ERK*, *PLCγ*, and *PI3K* pathways (30). Phosphorylation of *ERK* indirectly promotes phosphorylation of *CREB* through its downstream signaling molecules. Phosphorylated *CREB* binds onto the promoter region of *BDNF*, initiating its transcription. The *BDNF-ERK-CREB* pathway plays an important role in various neurobiological processes such as synaptic plasticity (31). Damage resulting from neuronal plasticity has been proven to be important in PSD pathogenesis (32). Numerous studies have further shown that reduced *BDNF* and *TrkB* are correlated to PSD (8,33). miR-22 was reported to inhibit *BDNF* expression by regulating translation of *BDNF* mRNA at the post-transcriptional level. An elevated miR-22 expression was found to inhibit the *BDNF-ERK-CREB* pathway, leading to PSD (34). In this study, we found an association between miR-22 and *TrkB* genes (*NTRK2*) following online prediction. Our prediction indicated that miR-22 might bind onto the 3′UTR region of target mRNA transcribed by *NTRK2*, inhibit synthesis of target protein (*TrkB*) and associated signal cascade reaction, destroy neuron plasticity, and eventually lead to PSD.

Previous animal studies have shown that PSD-related glucocorticoid receptor (GR) decreased in the frontal cortex and hippocampus (35,36). In addition, GR has been reported to inhibit expression of miR-22 by binding onto the transcription start site of miR-22 promoter (37). We hypothesized that PSD occurrence led to a decrease in GR and up-regulated expression of miR-22. Consequently, increased miR-22 expression could increase the risk of PSD, while occurrence of depression could also up-regulate miR-22 expression.

We also attempted to explore the possible mechanism of miR-22 action. Overall, we found that patients with brainstem and deep CMBs had a higher plasma miR-22 than the other group. This suggested that miR-22 was likely to be associated with brainstem and deep cerebral micro-vascular impairment, affecting the pathogenesis of PSD (P=0.028). A significant statistical result might be found by increasing the sample size. Vascular endothelial cadherin (VE-cadherin) is an important adhesion molecule, and plays a key role in maintaining the integrity of endothelial barriers, inflammation, and regulation of angiogenesis (38). Previous studies have shown that miR-22 binds to the 3′UTR region of VE-cadherin mRNA and regulates expression of VE-cadherin (10). Overexpression of miR-22 induced endothelial inflammation, damage to endothelial cells, increase in vascular permeability, destruction of blood-brain barriers, and led to abnormal angiogenesis and vascular dysplasia. On the other hand, inhibition of miR-22 expression protected vascular endothelial cells (10). Besides, damage to endothelial cells and blood-brain barriers played an important role in CMBs (39). Angiogenesis and myelin regeneration might maintain the stability and promote impairment of cerebral microvasculature through a coupling interaction (40). A high expression of miR-22 in PSD patients might lead to damage of endothelial cells, disrupt blood-brain barriers, interfere with endothelial-oligodendrocyte coupling, and cause impairment of cerebral microvasculature. It has further been shown that structural disruptions in the brainstem raphe and deep nerve pathways are accompanied by serotonergic transmission deficiency, which is eventually involved in the pathophysiology of PSD (23).

A number of limitations in our study are as follows: firstly, this was a single-center retrospective study with a limited sample size. It will be necessary to confirm these findings using large prospective clinical studies. Secondly, some patients with severe aphasia and severe disease were excluded from the study, which may introduce some bias in the observed results. Finally, during the 1-month period between MRI examination and depression evaluation, additional vascular events or other clinical events might have occurred, which could have affected the diagnosis of PSD.
